# Supplementary material for: A Human Organoid Model of Aggressive Hepatoblastoma for Disease Modeling and Drug Testing
Source: Cancers (Basel). 2020 Sep 18;12(9):2668. doi: 10.3390/cancers12092668 (PMC7563272; doi:10.3390/cancers12092668)
Supplement: Supplementary file 1 [file cancers-12-02668-s001.zip › cancers-907419-supply/Cancers-907419- supplementary.docx]

Supplementary Materials

**A Human Organoid Model of Aggressive Hepatoblastoma for Disease Modeling and Drug Testing**

James A. Saltsman, William J. Hammond, Nicole J. C. Narayan, David Requena,
Helmuth Gehart, Gadi Lalazar, Michael P. LaQuaglia, Hans Clevers and Sanford Simon


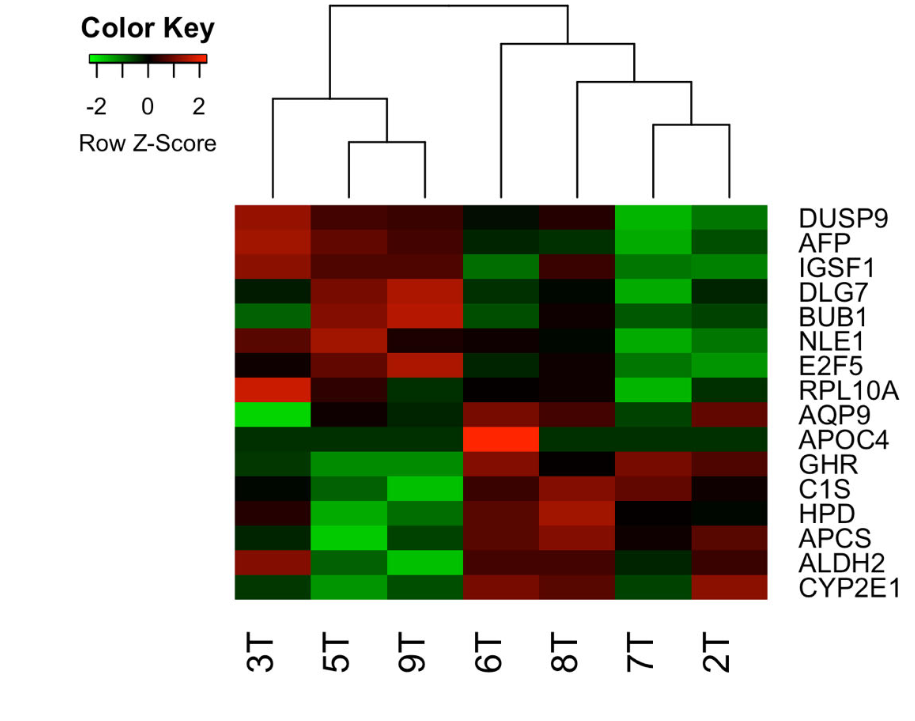


**Figure S1.** Cairo risk grouping for HB tumors.

**
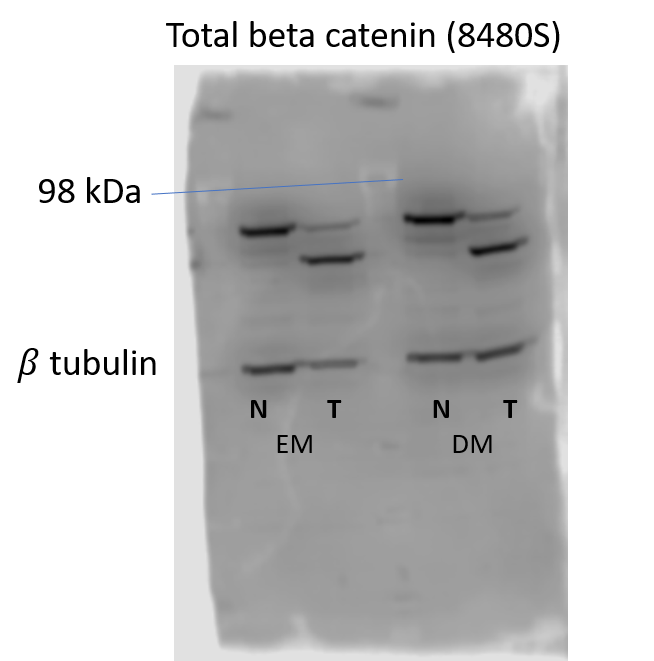

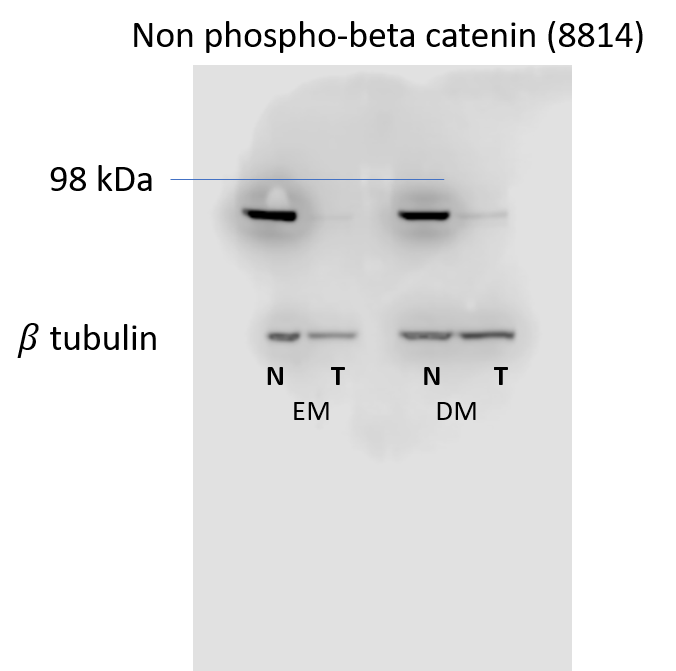

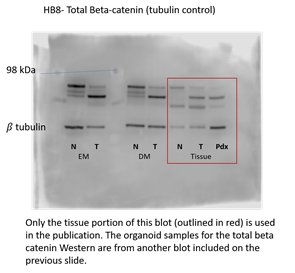
**


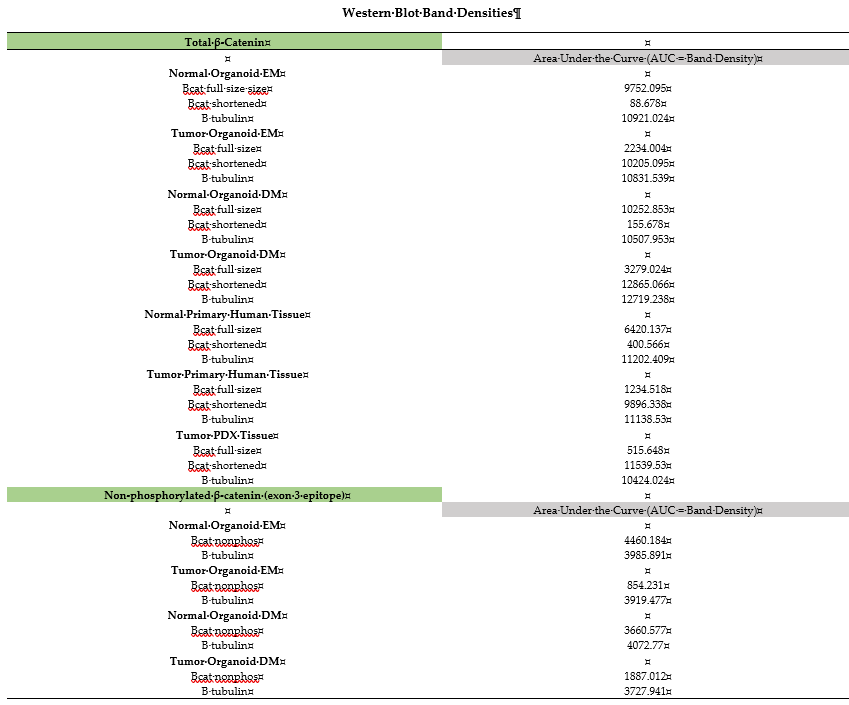


**Figure S2.** Detail information about Figure 4.

**Table S1.** Gene counts for Sumazin risk stratification.

| **Gene Symbol** | **LIN28B** | **HMGA2** | **AFP** | **SALL4** | **NFE2L2** |
| --- | --- | --- | --- | --- | --- |
| HB2N_Ti | 0.00 | 2.71 | 834.47 | 135.47 | WT |
| HB2T_Ti | 4.64 | 231.97 | 1,456.74 | 163.54 | WT |
|  |  |  |  |  |  |
| HB3N_Ti | 12.48 | 58.81 | 2,714.36 | 254.86 | WT |
| HB3T_Ti | 276.74 | 737.96 | 581,452.32 | 811.76 | WT |
|  |  |  |  |  |  |
| HB5N_Ti | 0.00 | 5.50 | 200.84 | 82.54 | WT |
| HB5T_Ti | 469.07 | 3,139.75 | 137,637.17 | 1,362.84 | WT |
|  |  |  |  |  |  |
| HB6N_Ti | 0.00 | 8.26 | 1,022.27 | 330.30 | WT |
| HB6T_Ti | 284.11 | 679.11 | 4,279.43 | 630.54 | WT |
| HB6N_DM | 1.52 | 272.57 | 40.53 | 11.15 | WT |
| HB6N_EM | 0.00 | 166.39 | 2.71 | 29.27 | WT |
|  |  |  |  |  |  |
| HB7N_Ti | 5.51 | 8.26 | 1644.12 | 137.70 | WT |
| HB7T_Ti | 9.38 | 32.05 | 137.59 | 87.56 | WT |
| HB7N_DM | 0.00 | 649.46 | 229.06 | 26.54 | WT |
| HB7N_EM | 1.02 | 74.44 | 0.00 | 57.11 | WT |
|  |  |  |  |  |  |
| HB8N_Ti | 0.00 | 11.23 | 886.83 | 164.64 | WT |
| HB8T_Ti | 142.87 | 354.33 | 3,056.07 | 527.20 | Mutated |
| HB8N_DM | 15.14 | 563.31 | 132.47 | 15.77 | WT |
| HB8N_EM | 0.00 | 205.69 | 1.07 | 23.09 | WT |
| HB8T_DM | 3332.18 | 1873.97 | 1932.77 | 83.06 | Mutated |
| HB8T_EM | 1951.87 | 870.81 | 2339.35 | 20.94 | Mutated |
| HB8T_PDX | 5703.52 | 73.69 | 203.59 | 879.92 | Mutated |
|  |  |  |  |  |  |
| HB9N_Ti | 33.23 | 71.21 | 557.31 | 18.99 | WT |
| HB9T_Ti | 5505.38 | 8403.54 | 65,462.76 | 1720.06 | WT |
| HB9T_PDX | 3258.33 | 5318.82 | 411,750.05 | 1456.74 | WT |

| 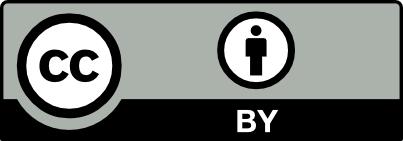 | © 2020 by the authors. Licensee MDPI, Basel, Switzerland. This article is an open access article distributed under the terms and conditions of the Creative Commons Attribution (CC BY) license (http://creativecommons.org/licenses/by/4.0/). |
| --- | --- |
